# Impact of COVID 19 on sleep quality: a study of 154 patients

**DOI:** 10.1192/j.eurpsy.2023.1660

**Published:** 2023-07-19

**Authors:** N. Halouani, D. Gdoura, A. Guermazi, M. Turki, N. Moussa, S. Ellouze, J. Aloulou

**Affiliations:** 1 Psychiatry “B” Departement; 2 Pulmonology Departement, Hedi Chaker university Hospital, Sfax, Tunisia

## Abstract

**Introduction:**

In addition to psychological distress, neurological and neurocognitive manifestations, the COVID19 pandemic and its medium- and long-term consequences combine other risk factors to alter sleep.

**Objectives:**

To screen for COVID19 sleep disorders and to identify epidemiological and clinical factors correlated with this disorder in post COVID19 patients.

**Methods:**

This is a descriptive and analytical cross-sectional study that took place during the period from the 1rst of March to the 15th of May 2021 with 154 patients who were hospitalized at the COVID unit19 at the Hedi Chaker Hospital in Sfax Tunisia.

The sleep evaluation, made by telephone, was performed using the “Insomnia Severity Index” scale.

**Results:**

The mean age was 66.62 ± 13.34 years. Male patients represented 60.4% of the study population.

In our study, the prevalence of anxiety, depression and post-traumatic stress disorder was 24.7%, 11% and 13.6% respectively.

The mean score of the sleep disorder severity index was 3.94 with extremes of score ranging from zero to 24. Thirty-six patients (23.4%) had insomnia, which was severe in 2.6% of patients.

We found a significant association between gender and sleep disorders. Thus, women were more likely to have insomnia.

A significant association was found between insomnia, anxiety-depressive disorders and post-traumatic stress disorder.

No significant association was found between disease characteristics and sleep disorders.

**Conclusions:**

In post COVID, patients suffer from an important sleep disorder. Indeed, the management of these sleep disorders in post Covid-19 is essential to improve the quality of life of these people.

**Disclosure of Interest:**

None Declared

